# Separating the direct effects of traits on atherosclerotic cardiovascular disease from those mediated by type 2 diabetes

**DOI:** 10.1007/s00125-022-05653-1

**Published:** 2022-02-07

**Authors:** Venexia M. Walker, Marijana Vujkovic, Alice R. Carter, Neil M. Davies, Miriam S. Udler, Michael G. Levin, George Davey Smith, Benjamin F. Voight, Tom R. Gaunt, Scott M. Damrauer

**Affiliations:** 1 MRC University of Bristol Integrative Epidemiology Unit, Bristol, UK; 2 Bristol Medical School: Population Health Sciences, University of Bristol, Bristol, UK; 3 Department of Surgery, University of Pennsylvania Perelman School of Medicine, Philadelphia, PA USA; 4 Department of Medicine, University of Pennsylvania Perelman School of Medicine, Philadelphia, PA USA; 5 K.G. Jebsen Center for Genetic Epidemiology, Department of Public Health and Nursing, NTNU, Norwegian University of Science and Technology, Trondheim, Norway; 6 Center for Genomic Medicine, Massachusetts General Hospital, Boston, MA USA; 7 Division of Cardiovascular Medicine, University of Pennsylvania Perelman School of Medicine, Philadelphia, PA USA; 8 Corporal Michael Crescenz VA Medical Center, Philadelphia, PA USA; 9 Department of Systems Pharmacology and Translational Therapeutics, University of Pennsylvania Perelman School of Medicine, Philadelphia, PA USA; 10 Department of Genetics, University of Pennsylvania Perelman School of Medicine, Philadelphia, PA USA; 11 Institute of Translational Medicine and Therapeutics, University of Pennsylvania Perelman School of Medicine, Philadelphia, PA USA

**Keywords:** Atherosclerotic cardiovascular disease, Coronary artery disease, Direct effect, Genome-wide association study, Indirect effect, Mediation, Mendelian randomisation, Peripheral artery disease, Type 2 diabetes

## Abstract

**Aims/hypothesis:**

Type 2 diabetes and atherosclerotic CVD share many risk factors. This study aimed to systematically assess a broad range of continuous traits to separate their direct effects on coronary and peripheral artery disease from those mediated by type 2 diabetes.

**Methods:**

Our main analysis was a two-step Mendelian randomisation for mediation to quantify the extent to which the associations observed between continuous traits and liability to atherosclerotic CVD were mediated by liability to type 2 diabetes. To support this analysis, we performed several univariate Mendelian randomisation analyses to examine the associations between our continuous traits, liability to type 2 diabetes and liability to atherosclerotic CVD.

**Results:**

Eight traits were eligible for the two-step Mendelian randomisation with liability to coronary artery disease as the outcome and we found similar direct and total effects in most cases. Exceptions included fasting insulin and hip circumference where the proportion mediated by liability to type 2 diabetes was estimated as 56% and 52%, respectively. Six traits were eligible for the analysis with liability to peripheral artery disease as the outcome. Again, we found limited evidence to support mediation by liability to type 2 diabetes for all traits apart from fasting insulin (proportion mediated: 70%).

**Conclusions/interpretation:**

Most traits were found to affect liability to atherosclerotic CVD independently of their relationship with liability to type 2 diabetes. These traits are therefore important for understanding atherosclerotic CVD risk regardless of an individual’s liability to type 2 diabetes.

**Graphical abstract:**

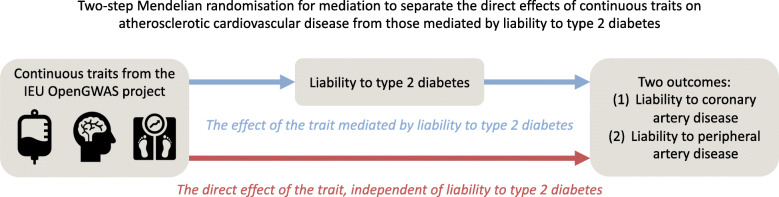

**Supplementary Information:**

The online version contains supplementary material available at 10.1007/s00125-022-05653-1.

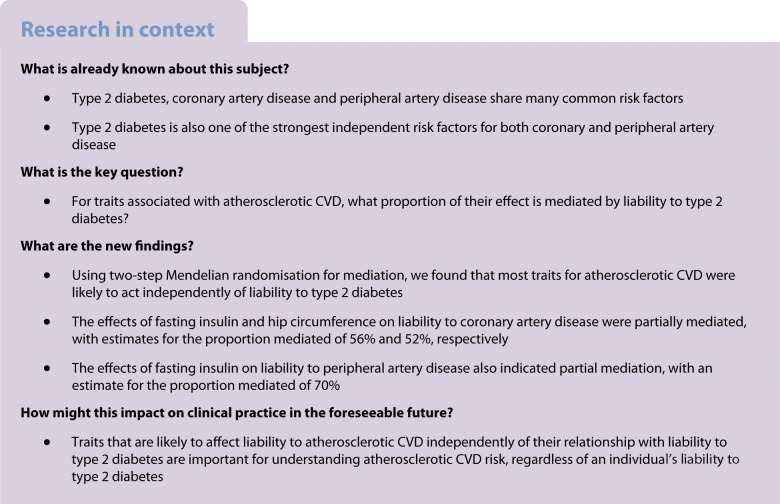



## Introduction

Type 2 diabetes shares several risk factors with the atherosclerotic CVDs coronary artery disease and peripheral artery disease. These risk factors include obesity and hypertension [1–3]. In addition, type 2 diabetes is one of the strongest independent risk factors for both coronary and peripheral artery disease [4, 5]. As a result of the shared links between type 2 diabetes and atherosclerotic CVD, it can be difficult to separate the direct effects of risk factors for atherosclerotic CVD from those mediated by type 2 diabetes. Distinguishing these effects is important because it may provide novel biological insight into the conditions individually, while also improving our understanding of their commonalities.

Mendelian randomisation uses genetic variants associated with an exposure (referred to as an ‘instrument’) as a proxy for that exposure [6]. This method can be used to estimate the causal effect of an exposure on an outcome free from bias due to non-genetic confounding and reverse causality if its assumptions hold [7]. Two-step Mendelian randomisation for mediation analysis is an extension to this method and incorporates the causal effect of a mediator, to estimate the direct (independent of the mediator) and indirect (via the mediator) effects of an exposure on an outcome [8, 9]. Furthermore, this approach can be applied using summary statistics from multiple genome-wide association studies (GWASs) with non-overlapping samples [10]. This removes the need for individual-level data from a single study containing information on all the risk factors, allowing broad systematic assessment of a wide range of risk factors unlikely to be captured in one place.

While Mendelian randomisation has previously been used to individually estimate the effect of several risk factors on liability to our three disease outcomes of interest [4, 11–15], and Mendelian randomisation for mediation has been conducted to investigate the mediating effect of a selected set of obesity-related markers [16], systematic assessment of a wide range of traits using Mendelian randomisation to separate their effects on liability to atherosclerotic CVD from liability to type 2 diabetes has not yet been conducted. The aim of this study was therefore to implement a standardised univariate Mendelian randomisation framework and follow-up analyses with two-step Mendelian randomisation for mediation to interrogate the association of a broad range of continuous traits with liability to our three disease outcomes: type 2 diabetes; coronary artery disease; and peripheral artery disease.

## Methods

### Study design

**Fig. 1:**
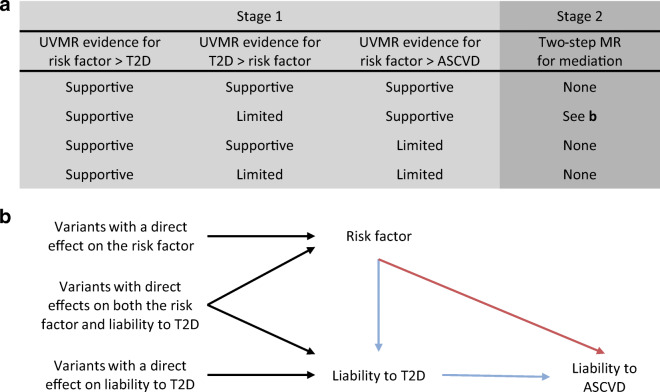
Illustration of the two-stage study design and the two-step Mendelian randomisation for mediation model used for stage 2. (**a**) Summary of how evidence from univariate Mendelian randomisation analyses of the risk factor, liability to type 2 diabetes, and liability to atherosclerotic CVD are assessed in stage 1. Here, estimates that met the arbitrary FDR threshold of 5% were deemed to lend ‘supportive’ evidence, while all other estimates were considered to provide ‘limited’ evidence. Depending on the evidence obtained in stage 1, a trait may progress to stage 2 (i.e. the two-step Mendelian randomisation for mediation). (**b**) The model for two-step Mendelian randomisation is shown. Red arrow represents the direct (i.e. independent of the mediator) effect and the blue arrow represents the indirect (i.e. via the mediator) effect. ASCVD, atherosclerotic CVD; T2D, type 2 diabetes; UVMR, univariate Mendelian randomisation; MR, Mendelian randomisation

Our study consisted of two stages (summarised in Fig. 1). First, we used univariate Mendelian randomisation to estimate the effects of 108 continuous traits (see Trait selection, below) on liability to three disease outcomes: type 2 diabetes; coronary artery disease; and peripheral artery disease. In addition, we used univariate Mendelian randomisation to estimate the effect of liability to type 2 diabetes on the 108 continuous traits. This allowed us to remove traits that had a bidirectional association with liability to type 2 diabetes as we cannot determine which phenotype should be the exposure and which the mediator for downstream analyses in this case. Based on the evidence from stage 1, we implemented stage 2: two-step Mendelian randomisation for mediation. Using this approach, we estimated the direct effect (i.e. independent of liability to type 2 diabetes) and indirect effect (i.e. mediated via liability to type 2 diabetes) of the traits on the atherosclerotic CVDs of interest.

### Trait selection

Traits were selected from the IEU OpenGWAS database by implementing a selection procedure to retain the largest, minimally adjusted GWAS for each continuous biological trait that had been studied in both men and women of European or mixed ancestry (electronic supplementary material [ESM] Fig. 1) [17]. Sample overlap was permitted between traits and so most of the GWASs included participants from UK Biobank [18].

### Outcome phenotypes

We obtained the GWASs for liability to type 2 diabetes in European ancestry from the DIAMANTE consortium [19]. The GWASs for liability to coronary artery disease and liability to peripheral artery disease were obtained from the CARDIoGRAM consortium and Million Veteran Program, respectively [20–22]. As noted above, sample overlap was permitted between traits, although GWASs were obtained from distinct samples for liability to type 2 diabetes, coronary artery disease and peripheral artery disease.

### Univariate Mendelian randomisation

Instruments for each trait were defined using the genome-wide significant (*p*<5 × 10^−8^) genetic variants from the corresponding GWAS to satisfy the first instrumental variable assumption of relevance. A description of relevance and the other the instrument assumptions required for Mendelian randomisation are given in ESM Methods 1. For the univariate Mendelian randomisation analyses, instruments were clumped using a 10 Mb window and *R*^2^ linkage disequilibrium (LD) threshold of 0.001 against the 1000 genomes reference panel for the European super-population, which was filtered to include only bi-allelic variants with minor allele frequencies greater than 0.01. Instruments consisting of less than ten variants were removed, before harmonisation with the outcome data to represent an increase in the exposure. Mendelian randomisation was then performed using the inverse variance weighted method. Note that all estimates are presented in SD units to allow comparison between traits.

We repeated the above univariate Mendelian randomisation analyses using the simple mode, weighted median, weighted mode and MR-Egger methods as a sensitivity analysis to examine estimate consistency. We also derived heterogeneity statistics to examine the consistency of estimates across the variants included in each analysis and performed a leave-one-out analysis to determine whether certain variants were driving the observed effects. We included an MR-Egger intercept test to assess whether directional pleiotropy was likely to have affected our results [23]. Finally, to assess the no measurement error assumption for MR-Egger, we calculated the ${I}_{GX}^2$ statistic as a measure of potential attenuation bias [24]. All univariate analyses and associated sensitivity analyses were implemented using the TwoSampleMR package for R [25].

### Two-step Mendelian randomisation for mediation

When we found the following evidence: (1) evidence to support an effect of the trait on liability to type 2 diabetes; (2) limited evidence to support an effect of liability to type 2 diabetes on the trait; and (3) evidence of an effect on liability to at least one atherosclerotic cardiovascular outcome of interest, multivariable Mendelian randomisation was applied using the trait and liability to type 2 diabetes as exposures. An arbitrary false discovery rate (FDR) of 5%, calculated according to the Benjamini and Hochberg method, was used as an indicator of supportive evidence of an association [26]. This multivariable Mendelian randomisation allowed us to estimate the effect of the trait, independent of liability to type 2 diabetes, on the liability to atherosclerotic CVD outcome of interest (Fig. 1b). This effect is often referred to as the ‘direct’ effect. We were also able to derive the effect of the trait, through liability to type 2 diabetes on liability to the atherosclerotic CVD outcome of interest, often referred to as the ‘indirect’ or ‘mediated’ effect. For the two-step Mendelian randomisation for mediation, we multiplied the estimate for the effect of the trait on liability to type 2 diabetes obtained from the univariate Mendelian randomisation by the direct effect of liability to type 2 diabetes on the atherosclerotic cardiovascular outcome of interest obtained from the multivariable Mendelian randomisation (where the exposure of interest and mediator were both used as exposures). CIs were derived using the sum of squares method.

Instruments for this analysis were clumped against either the trait or liability to type 2 diabetes (whichever had the smallest instrument) using a 10 Mb window and *R*^2^ LD threshold of 0.001 against the 1000 genomes reference panel for the European super-population, which was filtered to include only bi-allelic variants with minor allele frequencies greater than 0.01. Harmonisation was performed with variants aligned to represent an increase in the trait prior to analysis. We calculated conditional *F* statistics to test instrument strength for each exposure in our analysis. We also calculated a modified form of Cochran’s *Q* statistic that has been developed to measure heterogeneity in causal effect estimates from multivariable Mendelian randomisation. Multivariable Mendelian randomisation estimates and these statistics were obtained using the MVMR package for R [27]. Again, all estimates are presented in SD units to allow comparison between traits. The non-collapsibility of ORs can pose a problem when using summary statistics from logistic regression for binary mediators and outcomes in multivariable Mendelian randomisation. To assess whether this is likely to have impacted our results, we repeated our analyses using a GWAS of liability to type 2 diabetes based on a linear (instead of a logistic) model (ESM Methods 2). Finally, note that alongside the standard instrument assumptions required for Mendelian randomisation (ESM Methods 1), two-step Mendelian randomisation for mediation also assumes no interaction between the exposure and the mediator.

### Code availability

All analyses were conducted in R version 4.0.2. The associated code is available from https://github.com/venexia/T2DMediationMR.

### Ethics approval

This research using UK Biobank data was completed under Application Number 15825, which has been subject to ethics approval.

## Results

The results of this analysis are presented in four parts: (1) the selection of traits from the IEU OpenGWAS database [17]; (2) the results of the univariate Mendelian randomisation analyses to interrogate the effect of each trait on liability to type 2 diabetes and the effect of liability to type 2 diabetes on each trait; (3) the results related to liability to coronary artery disease from both the univariate Mendelian randomisation and two-step Mendelian randomisation for mediation; and (4) results related to liability to peripheral artery disease from the univariate Mendelian randomisation and two-step Mendelian randomisation for mediation.

### Trait selection

We identified 108 traits from the IEU OpenGWAS database for inclusion in our analysis [17]. Details of both the trait and outcome GWASs are provided in ESM Table 1. Most of the trait GWASs were conducted in UK Biobank by the Neale lab [28]. Twelve of the selected GWASs were from other sources: adiponectin [29]; alcoholic drinks per week [30]; body fat [31]; BMI [32]; cigarettes per day [30]; fasting glucose [33]; fasting insulin [33]; heart rate [34]; neuroticism [35]; total cholesterol [36]; urinary sodium-potassium ratio [37]; and waist/hip ratio [38].

### Causes and consequences of liability to type 2 diabetes

Estimates from bidirectional univariate Mendelian randomisation of each trait and liability to type 2 diabetes found evidence for ten traits as causes, but not consequences, of liability to type 2 diabetes at an FDR threshold of 5% (ESM Figs 2, 3; ESM Table 2). These traits were taken forward to the two-step Mendelian randomisation for mediation analyses for liability to atherosclerotic CVD. Sensitivity analyses using alternative Mendelian randomisation methods were consistent with the inverse variance weighted estimates (ESM Table 2). The MR-Egger intercept test found intercepts between −0.15 (body fat on liability to type 2 diabetes) and 0.07 (fasting glucose on liability to type 2 diabetes) (ESM Table 3). Finally, the ${I}_{GX}^2$ statistic was over 0.93 for all MR-Egger results (ESM Table 4). When taken as an estimate of the attenuation bias in these analyses, this corresponds to less than 7% relative bias towards the null.

### Causes of liability to coronary artery disease

**Fig. 2:**
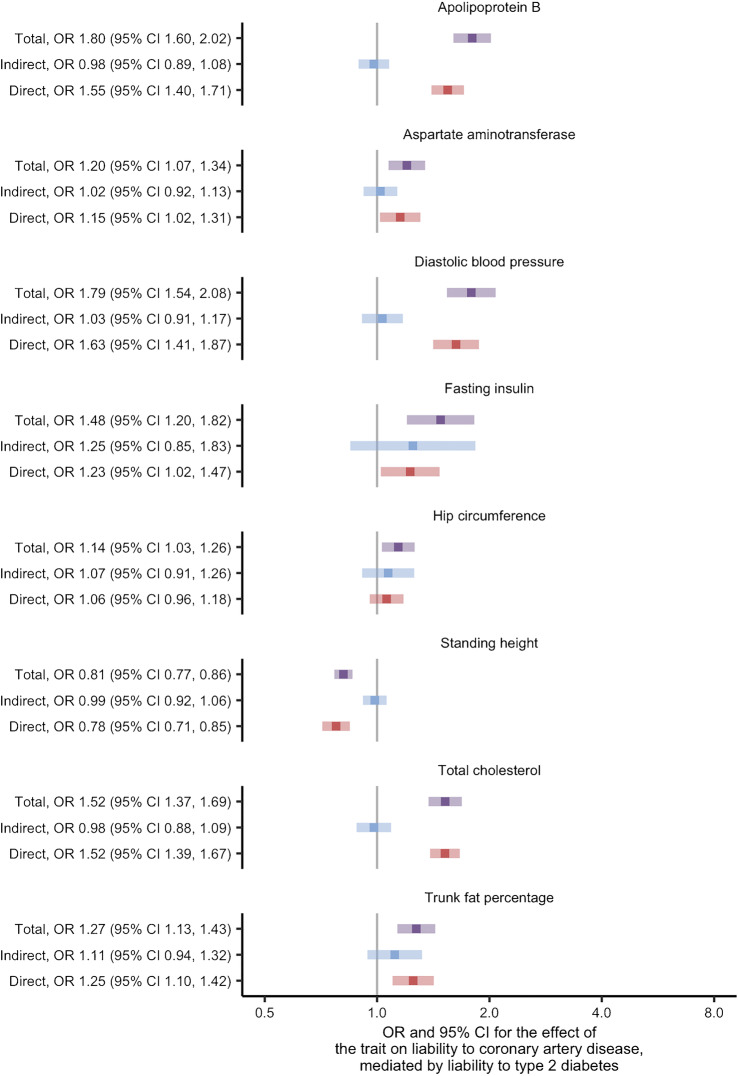
Two-step Mendelian randomisation for mediation estimates for the total, indirect (mediated by liability to type 2 diabetes) and direct (independent of liability to type 2 diabetes) effects of the indicated risk factors on liability to coronary artery disease

Using univariate Mendelian randomisation, we found evidence for 53 of the 108 traits as causes of liability to coronary artery disease at the FDR threshold of 5% (ESM Fig. 4; ESM Table 2). After restricting to traits thought to be causes of liability to type 2 diabetes, eight traits remained and were studied using two-step Mendelian randomisation for mediation (ESM Table 5, where variable ‘stage2_cad’ is true). In this analysis, we found similar direct and total effects for most traits: apolipoprotein B; aspartate aminotransferase; diastolic BP; standing height; total cholesterol; and trunk fat percentage (Fig. 2 and ESM Table 6). The exceptions were fasting insulin and hip circumference, where the effects indicated partial mediation by liability to type 2 diabetes. These effects corresponded to an estimate for the proportion of the effect mediated by liability to type 2 diabetes of 56% and 52%, respectively (ESM Table 7). The conditional *F* statistics for the multivariable Mendelian randomisation component of these analyses ranged from 9 to 87 (ESM Table 6), indicating good instrument strength. Meanwhile, the modified Cochran’s *Q* statistic exceeded the critical value for the χ^2^ distribution at the 5% level for all analyses. This indicated that the chosen SNPs predicted both the trait and liability to type 2 diabetes in the data. Taken as a whole, the analyses concerning liability to coronary artery disease suggest that the effects of the traits are likely to be independent of the effects of liability to type 2 diabetes.

### Causes of liability to peripheral artery disease

**Fig. 3:**
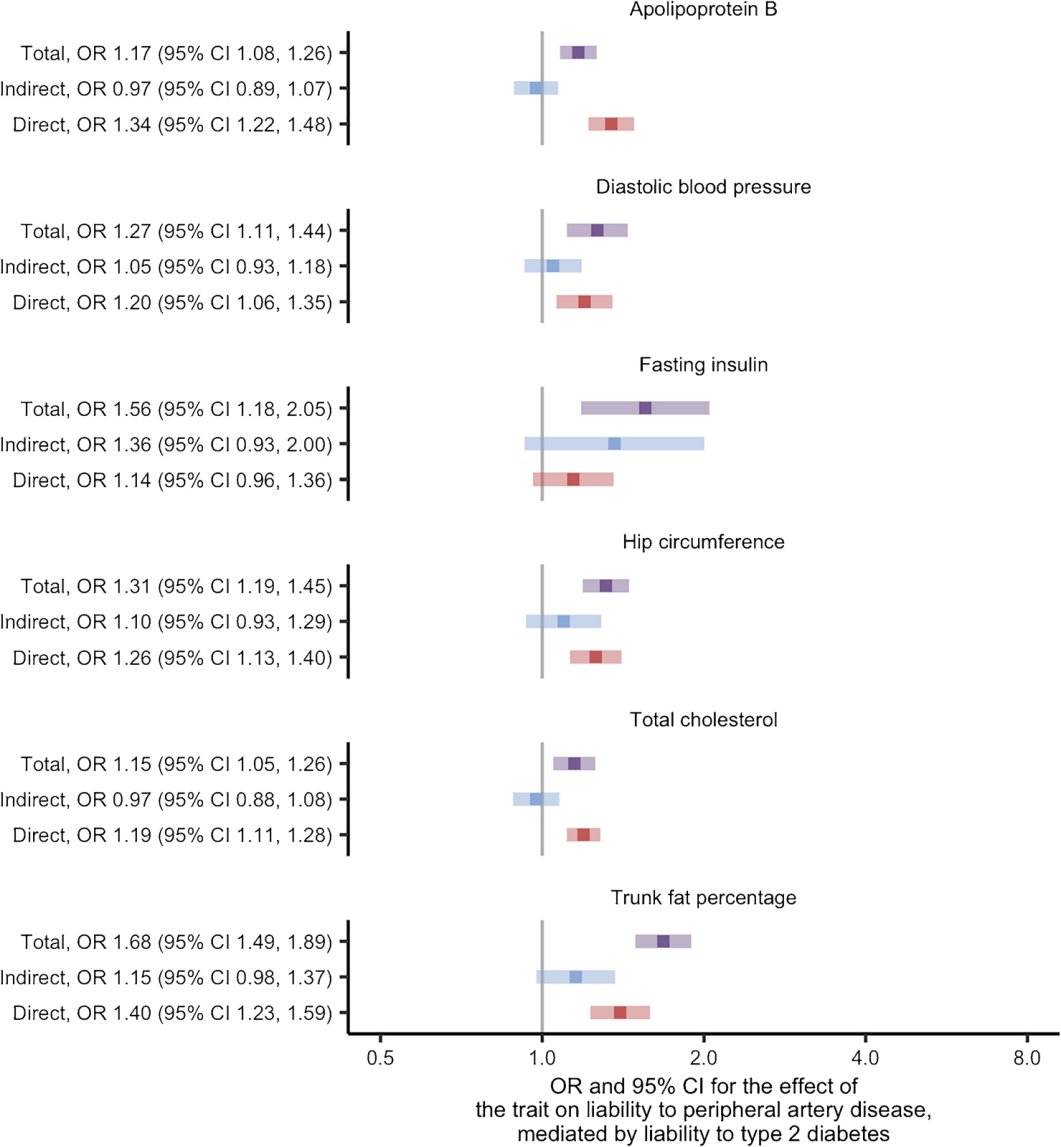
Two-step Mendelian randomisation for mediation estimates for the total, indirect (mediated by liability to type 2 diabetes) and direct (independent of liability to type 2 diabetes) effects of the indicated risk factors on liability to peripheral artery disease

We found evidence for 42 traits as causes of liability to peripheral artery disease at the FDR threshold of 5% using univariate Mendelian randomisation (ESM Fig. 5; ESM Table 2). After restricting to traits thought to be causes of liability to type 2 diabetes, six traits remained: apolipoprotein B, diastolic BP, fasting insulin, hip circumference, total cholesterol and trunk fat percentage (ESM Table 5, where variable ‘stage2_pad’ is true). Two-step Mendelian randomisation for mediation of these traits found similar direct and total effects in most cases (Fig. 3; ESM Table 6). Fasting insulin was again identified as an exception with effects that indicated partial mediation by liability to type 2 diabetes and an estimated proportion mediated of 70% (ESM Table 7). The conditional *F* statistics for the multivariable Mendelian randomisation component of these analyses again indicated good instrument strength, ranging from 9 to 86 (ESM Table 6). We also found the modified Cochran’s *Q* statistic exceeded the critical value for the χ^2^ distribution at the 5% level for all liability to peripheral artery disease analyses. Similar to the results concerning liability to coronary artery disease, these analyses suggest that the effects for most of the traits on liability to peripheral artery disease are likely to be independent of the effects of liability to type 2 diabetes.

## Discussion

**Fig. 4:**
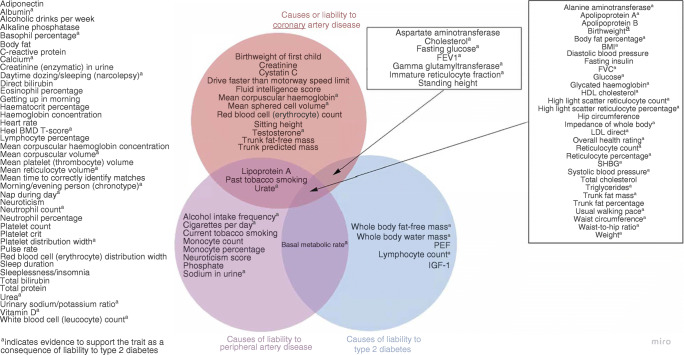
Venn diagram summarising the traits with evidence to support them as causes of liability to type 2 diabetes, coronary artery disease and peripheral artery disease. ^a^Evidence to support the trait as a consequence of liability to type 2 diabetes. BMD, bone mineral density; FEV1, forced expiratory volume in 1 s; FVC, forced vital capacity; PEF, peak expiratory flow; SHBG: sex hormone binding globulin

Using univariate Mendelian randomisation, we provide evidence for the causal effects of multiple traits on liability to our three outcomes of interest: type 2 diabetes; coronary artery disease; and peripheral artery disease, (Fig. 4). Common traits for liability to these outcomes included glycaemic traits such as glucose (type 2 diabetes, OR 3.34 [95% CI 2.41, 4.63] [ESM Fig. 2]; coronary artery disease, OR 1.25 [95% CI 1.11, 1.41] [ESM Fig. 4]; peripheral artery disease, OR 1.26 [95% CI 1.10, 1.44] [ESM Fig. 5]) and anthropometric traits such as body fat percentage (type 2 diabetes, OR 2.78 [95% CI 2.32, 3.32] [ESM Fig. 2]; coronary artery disease, OR 1.52 [95% CI 1.33, 1.73] [ESM Fig. 4]; peripheral artery disease, OR 1.92 [95% CI 1.68, 2.19] [ESM Fig. 5]). We also identified specific traits for each outcome. For instance, there were five traits with evidence to support an effect on liability to type 2 diabetes (whole-body fat-free mass, whole-body water mass, peak expiratory flow, lymphocyte count, IGF-1) but not liability to coronary or peripheral artery disease, as well as 12 and eight traits with specific effects on liability to coronary and peripheral artery disease, respectively. These findings confirm several known traits for each of the outcomes and may provide novel biological insight regarding some of the lesser-studied traits.

Using two-step Mendelian randomisation for mediation analysis, this study found that the effects of most of the eligible traits were likely to be independent of the effects of liability to type 2 diabetes. There are several reasons why a mediating effect may not have been identified in this analysis. First, there could be no true mediating effect, so our findings reflect reality. Second, we may lack power to detect a mediating effect as the power requirements for multivariable Mendelian randomisation are greater than univariate approaches and the number of traits considered in this study comes with a high multiple testing burden. Alternatively, the phenotypic complexity of liability to type 2 diabetes may be obscuring effects if, for example, a trait acts on a certain component of liability to type 2 diabetes that does not have a causal effect on liability to atherosclerotic CVD. Partial mediation was observed for two traits: fasting insulin, which is difficult to separate from the clinical definition of type 2 diabetes, and hip circumference, though this particular trait was only an exception for the outcome liability to coronary artery disease. Several of the traits tested, including BMI and waist/hip ratio, were identified as both causes and consequences of liability to type 2 diabetes and so were not studied using two-step Mendelian randomisation for mediation, even if the magnitude of the effects heavily favoured a direction. Despite this, the strong causal effects observed for these traits on liability to coronary and peripheral artery disease, without consideration of liability to type 2 diabetes, indicate that they remain important traits for reducing the risk of atherosclerotic CVD outcomes.

Four traits included in this study may be considered as part of the clinical definition of type 2 diabetes or ‘endophenotypes’ of type 2 diabetes, namely fasting glucose, fasting insulin, glucose and HbA_1c_. Except for fasting insulin, which was found to be a cause but not a consequence of liability to type 2 diabetes, these traits were deemed to have bidirectional relationships with liability to type 2 diabetes when interpreted using the arbitrary 5% FDR threshold selected for this study. Given the interrelated nature of these glycaemic traits with liability to type 2 diabetes, this is unsurprising and highlights the difficultly in disentangling these effects. Nonetheless, it was important to include these traits in our analysis given our aim of systematically assessing the effects of traits on liability to atherosclerotic CVD risk.

Biologically, our results highlight the centrality of glycaemic traits in the shared causal pathways between type 2 diabetes and CVD, and in mediating the effect of type 2 diabetes on atherosclerotic CVD. There is evidence in the literature to suggest that glycaemic traits may have direct effects on atherosclerotic CVD that are independent of liability to type 2 diabetes. For instance, insulin sensitivity has been shown to be a marker of coronary artery disease risk in non-diabetic populations [39, 40]. Similarly, human genetic evidence suggests that average blood glucose levels linearly influence CHD risk even within the physiologically normal range [41]. Although focus on glycaemic control has variable effects on CVD outcomes among large cohorts of individuals with diabetes, in sum, these findings suggest that glycaemic traits play a key role in the development of atherosclerotic CVD [42, 43].

From a clinical standpoint, our results help clarify the goals of risk factor modification for the prevention of type 2 diabetes, as well as coronary and peripheral artery disease in the setting of type 2 diabetes. They suggest that although diabetes and atherosclerotic CVD share several risk factors, the effects of these traits on atherosclerotic CVD are independent. Clinically, these findings argue for broad risk factor modification, rather than targeting glycaemic control as the sole mediator of atherosclerotic CVD risk in individuals with type 2 diabetes. These findings fit within a broader clinical framework built on lifestyle modification, whereby dietary and exercise interventions may influence several cardiovascular risk factors simultaneously, including diabetes/glycaemic traits, obesity, BP and dyslipidaemia.

Our study has some limitations. Mendelian randomisation requires several assumptions to hold for valid estimates to be obtained and two-step Mendelian randomisation for mediation further requires no interaction between the exposure and mediator [6, 7]. Except for relevance, these assumptions cannot be tested. However, where possible, we have performed sensitivity analyses and falsification tests. In addition, our study may be subject to weak instrument bias as a small number of instruments have *F* statistics that fall below the common (arbitrary) threshold of 10. We report these results, with their *F* statistics, but encourage readers to be cautious in the inferences made from these estimates. Our study may also be biased due to the non-collapsibility of ORs, which can impact estimates as a result of summary statistics from logistic regression being used for binary mediators (such as liability to type 2 diabetes) and outcomes (such as liability to coronary and peripheral artery disease) [9]. We assessed this possibility by repeating our analyses with summary statistics from a novel GWAS that used a linear model for liability to type 2 diabetes and found little difference in the Mendelian randomisation estimates we obtained (ESM Methods 2; ESM Fig. 6). This indicates that non-collapsibility of ORs is unlikely to have impacted our results. In addition, our study may be affected by horizontal pleiotropy. We used MR-Egger estimators to investigate whether our results were sensitive to assumptions about the structure of pleiotropy and found some evidence that a small number of traits may have horizontally pleiotropic effects. Finally, our study was restricted to individuals of European or mixed ancestry due to the broad range of GWAS required for the analysis. Consequently, the generalisability of the findings from this study is limited to comparable European or mixed ancestry populations.

In conclusion, we have used a Mendelian randomisation framework to separate the effects of continuous traits from liability to type 2 diabetes and aid our understanding of their relationships with liability to coronary and peripheral artery disease. Our analysis suggests that some key traits, including diastolic BP and hip circumference, act independently of liability to type 2 diabetes. These traits are therefore important for understanding atherosclerotic CVD risk regardless of an individual’s liability to type 2 diabetes.

## Supplementary information

ESM 1 (PDF 2979 kb)

ESM 2 (XLSX 224 kb)

## Data Availability

All data used in this study are publicly available. We accessed genome-wide association study summary statistics for the traits from the IEU OpenGWAS database (https://gwas.mrcieu.ac.uk/), for liability to type 2 diabetes from the DIAMANTE consortium (https://www.diagram-consortium.org/), for liability to coronary artery disease from the CARDIoGRAM consortium (http://www.cardiogramplusc4d.org/) and for liability to peripheral artery disease from dbGAP (https://www.ncbi.nlm.nih.gov/gap/). Approved dbGAP access to phs001672.v6.p1 was provided to BFV (dbGAP project ID: 27398).

## Abbreviations

FDR: False discovery rate
GWAS: Genome-wide association studies

## Code availability

All analyses were conducted in R version 4.0.2. The associated code is available from https://github.com/venexia/T2DMediationMR.
